# Report of the Chicago Dental Society

**Published:** 1865

**Authors:** Jas. C. Dean


### REPORT OF THE CHICAGO DENTAL SOCIETY.

BY JAS. C. DEAN.

The regular monthly meeting of the Chicago Dental Society was held May 8th, at S. S. White's Dental Depot. President Geo. H. Cushing in the chair. After the regular business, the essayist for the evening, Dr. Dean, read a paper on filling teeth, at the conclusion of which Dr. Ellis moved a vote of thanks to the essayist, for his very acceptable paper; pending which the paper was very thoroughly discussed.

Dr. Ellis said our failures are too little discussed or brought forward; has had severe trials the last two weeks with one of those very exceptional cases that are occasionally brought forcibly to our notice. Patient, a young lady about 16 years, teeth badly decayed and crowded, could not keep patient in position over a minute or two at a time, could not put a napkin in the mouth on account of producing nausea, tongue uncontrolable, &c. Would like to see any of those dentists who proclaim at conventions and elsewhere, or through public journals, that they can always, without fail, keep the mouth dry and control patients at will, take that case.

Thinks filling can not all be done by rule and theory; often has a case that tries a man's resources, and calls for the very best skill and inventive faculty. Uses amalgam, has never seen any bad results from its use. Likes rapid wedging and a great deal of it. Uses the mallet and thinks it saves time and labor, also, its use is easier for both patient and operator.

Dr. Fuller prefers rubber for separating teeth, uses it about twenty-four hours and then inserts a fine wedge. Fills sensitive teeth with Hill's stopping, letting it stay in the cavity from one to six months or longer, and has good success in accomplishing the object.

Dr. Freeman prefers cotton for separating teeth, and thinks there is much less inflammation attending its use than by the use of rubber. Has used Wood's metal in teeth in the mouth once or twice, but don't like it.

Dr. Dean suggested that Dr. F. had not sufficient practice to enable him to use it effectively or successfully.

Dr. Sherwood thinks the efficacy of the file is in danger of being overlooked; had his teeth, all the anterior ones, filed apart to the extent of one-third of the substance of the crown twenty-nine years ago by Dr. Bigelow of New York and they have been thoroughly preserved since and are good to this time. By request, gave the members present an opportunity of seeing them, and demonstrating in his case that filing had not injured his teeth except as to shape. Uses soft foil principally more than adhesive, thinks there have been more bad fillings since adhesive foil came into use, or more particularly sponge gold, within the last five years, than in fifteen years, when soft foil was used exclusively. Uses amalgam of the precipitated silver and mercury and squeezes out the mercury through buck skin, with pliers washes out the oxide with alcohol.

Dr. Cushing wedges very gradually with pine; uses adhesive gold almost entirely, it must be thoroughly packed. Uses amalgam occasionally, but never likes to, only when nothing else could be used; thinks the quick wedging rather severe, and patients prefer the more gradual way.

Dr. Dean agreed with Dr. Ellis that a good dentist must be skillful and capable of devising ways and expedients for the very many different opperations that are constantly presenting themselves, and referred to the same idea as presented in his essay. Was glad the essay had called forth such an interesting discussion and thereby accomplished the end sought to be obtained by it. Uses annealed gold almost entirely and referred again to his essay to show that in advocating annealed gold, he made a point therein that it must always be thoroughly packed. Likes the quick wedging if the patient will endure it; also thinks that there will be less inflammation attending it. Thinks an anæsthetic influence may be had on the nerves of the teeth thus separated by a sort of strangulation, the nerves being confined between the edges of the foramen at the apex of the root, and the edges of the entrance of the nerve canal at the bottom of the socket, and the said edges pressing toward each other and preventing sensation from being conveyed through the foramen at the apex. Likes Wood's metal very much, has used it about two years and substitutes it for amalgam whenever it can be done. Has had such patients as described by Dr. Ellis and thinks also with him, that patience almost ceases to be a virtue under such circumstances.

The question was then put and unanimously passed.

Dr. S. S. White of Philadelphia was present, and was called on for some remarks or words of encouragement, and kindly did so from the text, "it is more blessed to give than receive," and showed from past experience, that those dentists who had given freely had not suffered thereby, but had progressed not only in temporal goods, but also in the estimation of the profession generally.

(The secretary regrets that owing to the great interest he took in the excellent remarks of Dr. White he omitted or found it impossible to make an adequate report of them.)

Dr. White recommends that the secretary should take notes of the remarks of each member and request each one, at an early period after the meeting, to make a concise report of them from said notes, furnished by the secretary, so as to enable him to make a report to be published in the dental journals.

